# Career Development, Institutional Factors, Social Factors and Urban Young Returnees’ Happiness in the Context of Healthy China

**DOI:** 10.3390/ijerph19159379

**Published:** 2022-07-31

**Authors:** Feiwei Shen, Jing Zou, Xianhong Huang, Cong Wang, Mingjie Zhao

**Affiliations:** 1 College of Public Administration, Hangzhou Normal University, Hangzhou 311121, China; zjsfw.hi@163.com; 2 School of Public Affairs, Zhejiang University, Hangzhou 310058, China; 3 Hangzhou International Urbanology Research Center and Zhejiang Urban Governance Studies Center, Hangzhou 311121, China; 4 School of Finance, Zhejiang University of Finance and Economics, Hangzhou 310018, China; zhaomj00@163.com; 5 Department of Health Policy and Management, School of Public Health, Hangzhou Normal University, Hangzhou 311121, China; hxh974291@163.com; 6 School of Public Health, Hangzhou Normal University, Hangzhou 311121, China; wang11172021@163.com

**Keywords:** urban young returnees, happiness, healthy China, HLM, SEM

## Abstract

In the context of the Healthy China 2030 Plan, the importance of the happiness of urban young returnees should not be underestimated. Based on a large-scale social survey of social practices in China, this paper applies a hierarchical linear regression model (HLM) and a structural equation model (SEM) to investigate the determinants of urban young returnees’ happiness. The results show that the happiness of urban young returnees in China is not only influenced by their socio-demographic characteristics, such as age and education, but mainly by their occupational development, institutional factors (especially the employment and entrepreneurship policy system) and social factors (physical environment and urban rural relationship), which are different from those of ordinary residents. Further study shows that occupational development indirectly affects the happiness of urban young returnees through relationship adaptation, collective adaptation and material adaptation, the indirect effects accounts for 42.18%, 21.64% and 36.18%, respectively. Institutional factors exert an indirect effect on the happiness of urban young returnees through relationship adaptation (46.80%) and material adaptation (53.20%). Social factors indirectly affect the happiness of urban young returnees through relationship adaptation (44.20%), collective adaptation (16.96%) and material adaptation (38.84%). Policies to improve the happiness of urban young returnees are suggested.

## 1. Introduction

Health is an indispensable condition for the comprehensive development of people and a basic condition for economic and social development. Today, countries around the world are gradually regarding improving people’s quality of life and health as their top priority, and doing so has become a popular topic of research. Since 2014, China has gradually attached importance to “national health,” which has become an important part of the national strategy. The outline of the Healthy China 2030 plan issued by the CPC Central Committee and the State Council proposes promoting the equalization of basic public health services; focusing on rural areas and grassroots units; gradually reducing the disparities in basic health services and health levels between urban and rural areas, regions and people; and promoting social equity. In improving the levels of basic public services in rural areas, we need to comprehensively promote the construction of healthy villages. Urban young returnees are the backbone of healthy rural construction, so their sense of happiness should not be ignored. Numerous studies focus on factors affecting people’s happiness, such as socio-demographic characteristics [1], economic factors [2], social capital [3], social trust [4], migration and neighbourhood characteristics [5], but ignore the happiness of young returnees, especially the institutional and social factors in China. Due to the gap between urban and rural areas in terms of economy, culture and other aspects, urban young returnees have different degrees of psychological imbalance in rural life, which leads to short-term and long-term adaptation obstacles, thus affecting their happiness.

To fill the research gap, this paper contributes to the literature in three important ways. First, the particular advantage is that our unique data allow us to explore the determinants of urban young returnees’ happiness in the context of a healthy China, where urban young returnees are a specific group that that does not receive enough attention. Second, HLM and SEM are applied to analyse the influencing factors of urban young returnees’ happiness. Third, social adaptation theory is used to construct the theoretical framework, and material adaptation, collective adaptation, relationship adaptation and psychology adaptation are introduced as the underlying mechanisms.

### 1.1. Literature Review

#### 1.1.1. The Concept of Urban Young Returnees and Happiness

With the increase of urban young returnees, increasing numbers of scholars are addressing them. Urban young returnees are usually defined as individuals who return to their hometown or other former place of residence. They are an important component of adult migration in the late 20s and 30s [6]. Happiness at the individual level has been studied and defined in several ways, for instance referring to individuals’ overall evaluations of their lives [7] including their utility and subjective welfare [8]. There are a number of characteristics associated with happiness such as subjective well-being [9] and life satisfaction [10]. Happiness is a “positive emotional state”, which expresses something more enduring than simply “feeling good”, extending to a tendency toward positive feelings and moods [11]. In this framework, saying one is happy implies a general “psychic affirmation” of one’s life. In terms of measuring happiness, some studies use a single question, such as, Overall, do you feel happy with your life? [12] Some studies use factor analysis to construct the feeling of happiness [13]. In this paper, in combination with the questions in the survey, the feeling of happiness was measured in four dimensions using factor analysis: (1) My life is close to my ideal in many ways. (2) My goal in life is enough to give me the motivation to struggle. (3) I feel happy when I devote myself to what I do. (4) I think my work can bring positive effect to the development of my hometown.

#### 1.1.2. Previous Studies on Determinants of People’s Happiness

Happiness, as a critical goal of human life, has been widely explored in the fields of psychology, sociology, economics and others [3,14]. At the same time, the determinants of happiness have also been discussed.

Previous research mainly focuses on socio-demographic factors affecting people’s happiness such as gender, age, education and marital status. Some studies suggest that females are happier than males [15], while other studies show that there is no difference in happiness between men and women [16]. Some researchers concluded that age does not impact happiness [16], while others established a U-shaped relationship between age and happiness [17]. In general, married people are happier than single people [15,16]. Regarding education, there is no consensus. Some found that education is positively related to happiness [15,16], while others determined that the relationship is negative [18]. However, the personal factors mentioned above can only explain a small part of the differences [19].

The associations between economic and health factors and happiness has also been discussed. Most scholars believe that there is a positive but weak relationship between income and happiness [20]. However, some studies show that income is negatively associated with happiness [21,22]. If income matters mainly in a relative sense [23], migration to a wealthier country could hinder one’s happiness, and unemployment can also have a negative impact on well-being [18]. However, health has a positive effect on happiness [24].

Social relationships are also an essential element that influences happiness [20,25,26]. Reflix (2003) [27] defines these as cooperative relationships among social actors promoting collective action. Their core components are civic participation and mutual trust among community members. People who surround themselves with friendly, helpful and trustworthy neighbours are happier than lonely people [28]. Social interaction with neighbours can promote people’s happiness. In addition, socio-cultural features are associated with well-being [29], and the emotional experience of daily activities also has a significant impact on people’s happiness [30]. Using an instrumental variable and two-stage residual inclusion, Lu et al. (2019) confirm the positive relationship between social trust and happiness in China [4].

The relationship between migration and happiness is also an important issue. The most commonly used method of measuring this relationship is to compare the happiness of migrants with that of local residents, and the common finding is that migrants are less happy than natives [31]. Bartram (2013) compares migrants with those who stayed in the country they left, and he finds that migrants from eastern to western Europe do not experience greater happiness [32]. However, for migrants from countries where the average happiness level is low, migrants seem to become happier after moving. Later, he finds that migrants are less happy than stayers when they move from wealthier to poorer countries in Europe [2]. On the contrary, migrants from Germany are happier than those who stay in Germany. Longitudinal analysis of the panel data indicates that migration to wealthier areas has a positive impact on happiness [33].

Until recently, some scholars have concentrated on the relationships between housing, neighbourhood and happiness, primarily examining the community’s social attributes as the underlying mechanism. For example, residents feel happier in less deprived communities [20]. Community influences life satisfaction through community satisfaction, while community economic characteristics influence life satisfaction through housing and family satisfaction [34]. In addition, the community’s social composition, such as race and ethnicity, can also affect residents’ happiness. For instance, homogeneous groups are beneficial for the happiness of the people who live in them [1,35]. In China, most research focuses on the association between homeownership and happiness and finds that homeowners are happier than tenants [36,37,38]. In addition, Chen et al. (2021) find that Chinese urban residents’ happiness is higher when the local government promises greater commitment to improving housing affordability [39]. Few studies focus on the role of the community, but using Guangzhou survey data, Liu et al. (2017) hypothesize that neighbourhood relationships directly improve migrants’ happiness and that the correlation is more robust for locals than for migrants [40]. Migrants living in commercial housing, work unit housing, affordable housing and urban villages are happier than those living in old housing communities [5].

In summary, most research focuses on the determinants of people’ happiness that arise from personal characteristics, economic factors, health status, social relations, migration, housing and community characteristics, but the factors that influence urban young returnees’ happiness are ignored. As a very special group, urban young returnees come to the city and then return to the countryside and have experienced the dual influences of urban and rural life. Naturally, their psychological state is quite different from that of ordinary residents, and their adaptability to return to the countryside and their happiness also need to be reconsidered. Therefore, this paper will concentrate on this issue and explore the internal path of urban young returnees’ happiness, so as to improve their well-being and present suggestions for the healthy China strategy.

### 1.2. Theoretical Framework

According to the seventh census published by the National Bureau of Statistics in 2021, there are more than 375 million migrants in China. More and more migrants are returning to their hometowns. In 2020, there will be 10.1 million entrepreneurs and innovators returning home. That is 1.6 million more than in 2019, the year with the largest and fastest growth in recent years. Returning youth have gone through two stages: rural to urban and urban to rural. The main reason for returning is the difficulty of finding a job [41], followed by family reasons such as caring for family members [42]. In addition, community and macro political factors affect returning behaviour [43].

**Figure 1:**
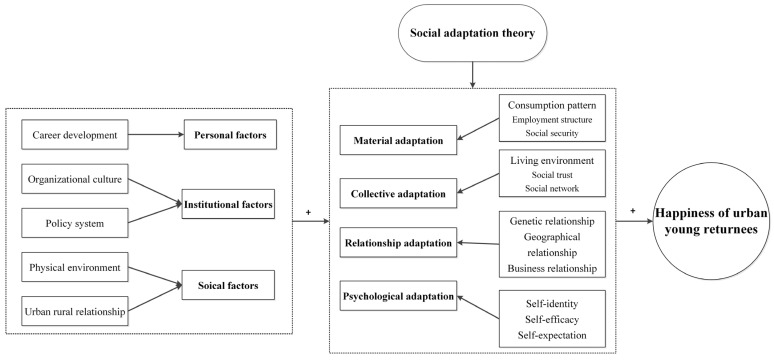
The theoretical framework of the study. Source: Authors.

As an amphibious group living in both the city and the countryside, urban young returnees vacillate between putting down roots in the city and returning to the countryside. Rural society has its own way of living working and communicating, as well as its own logic of living, surviving and operating. Today, great changes have taken place in rural areas, and the country attaches great importance to rural revitalization. There are both opportunities and challenges for urban young returnees returning to the countryside. When the urban young returnees return to the countryside, they may be confronted with the great divide between urban and rural life and the weakening of their past relationships with the countryside. Social adaptation is the process by which individuals’ concepts and behaviours change with the social environment in order to adapt to it [44], and urban young returnees should constantly adjust their subjective adaptation to the rural society to improve their sense of happiness. Different scholars distinguish different types of social adaptation, including occupational adaptation [45], economic adaptation [46], social interaction [46], psychological adaptation [47] and cultural adaptation [48]. Here, based on the information in the questionnaire, social adaptation is divided into four types by factor analysis, including material adaptation, collective adaptation, relationship adaptation and psychological adaptation. Among them, material adaptation includes consumption patterns, employment structure and social security; collective adaptation includes living environment, social trust and social network; relationship adaptation contains genetic relationship, geographical relation and business relationship; and psychological adaptation refers to self-identity, self-efficacy and self-expectation. This should summarize the various adaptations of today’s urban young returnees to the rural areas. The personal factors (career development), institutional factors (organizational culture and policy system) and social factors (physical environment and urban–rural relationship) perceived by urban young returnees will change and adjust through their various social adaptations and ultimately affect their happiness, and all the effects may be positive. The theoretical framework is proposed in Figure 1.

## 2. Materials and Methods

### 2.1. Data

The data are from a large-scale professional social survey conducted by Hangzhou Normal University in January to February 2021. The respondents (urban young returnees) are residents registered in rural areas who have chosen to return to the countryside as their main place of production and living after a period of urban life, including migrant workers, entrepreneurs, Xin Xiangxian (people who have a love of hometown in contemporary villages, who once went away for an official reason but returned to the countryside to teach or took root in the countryside for a long time and served the countryside with their own knowledge and ability), young college students and retired servicemen. In conjunction with the theoretical framework and a literature review, preliminary questionnaire items were drawn up, and 60 urban young returnees were surveyed. Then, the reliability and validity of the data were tested, and the formal questionnaire was adjusted according to the results. Based on the large-scale special social practices organized by Hangzhou Normal University, nearly 800 volunteers were recruited to conduct research in four regions of east, middle, west and northeast China.Probability proportionate to size sampling (PPS) was used to select the samples. A total of 3140 questionnaires were sent out, and 2234 questionnaires were returned, representing a recovery rate of 71.1%. After eliminating the questionnaires with obvious rules and inconsistent answers, a total of 2202 valid questionnaires were obtained, an effective rate of 98.6%.

Table 1 displays the basic information of the respondents. Most urban young returnees are between 21 and 40 years old, about 55.2% of them are female and 56.3% of them have a bachelor’s degree. The proportion of single urban young returnees accounts for 57.4%, and 56.4% of urban young returnees stay in the city for 3–5 years. Another 28.6% of them return for more than 5 years, while 21% of them return for 1 to 2 years. Most urban youth have no children or only one child. Comparing the change in salary between before and after returning to the hometown, the current income is higher than the previous income in the city. Looking at the change in occupation before and after returning to the hometown, the ratio of unemployed decreased significantly, from 33.9% to 15.7%. The proportion of urban young returnees engaged in science, education, culture, health and social security increased significantly, from 17.9% to 30.7%. The proportion of urban young returnees engaged in agriculture, forestry, husbandry and fishery also increased, from 4.3% to 8.6%.

### 2.2. Variable

According to the theoretical framework, we identified the antecedents, mediators and outcome variables of this study. The antecedent variables include individual factors, institutional factors and social factors, the intermediary variables include individual adaptation, relationship adaptation, collective adaptation and material adaptation, and the outcome variable is the happiness of urban young returnees. Their specific meaning and the results of confirmatory factor analysis conducted using Amos software are shown in Table 2. According to the results, Cronbach’s α are all above 0.9, indicating that the scale has good reliability. KMO and AVE were all above 0.7, indicating that the scale has good validity.

### 2.3. Methodology

First, to analyse the differences in happiness among urban young returnees in China, multivariate analysis of variance and *t* test are applied. In addition, correlation analysis is used to analyse the association between the independent variables and the happiness of urban young returnees.

For non-equilibrium nested data, the hierarchical linear model can decompose and estimate the variance and covariance components in the data and reveal their hierarchical differences. Therefore, this paper adopts the HLM model as the basic research method, and the specific settings of this model are as follows:

(1) $$Happiness_{i}=\beta_{0}+\beta_{1}Socio\_demo_{i}+\varepsilon_{i}$$

(2) $$Happiness_{i}=\beta_{0}+\beta_{1}Socio\_demo_{i}+\beta_{2}Per_{i}+\beta_{3}Inst_{i}+\beta_{4}Soci_{i}+\varepsilon_{i}$$

(3) $$Happiness_{i}=\beta_{0}+\beta_{i}X_{i}+\varepsilon_{i}$$

Among them, $Happiness_{i}$ is the happiness of urban young returnees. $Socio\_demo_{i}\;$is the social demography factors, including gender, age, education, returning time, current occupation and current monthly income. $Per_{i}$ means personal factors such as career development. $Inst_{i}\;$is institutional factors, containing organizational culture and policy system. $Soci_{i}$ represents the social factors, including physical environment and urban rural relationship. $X_{i}$ represents all the control variables. $\varepsilon_{i}$ is the random error term.

However, among the variables in this study, individual factors, institutional factors and social factors of urban returnees cannot be measured with a single index, and happiness refers to the subjective understanding and willingness of urban returnees and cannot be directly measured. These characteristics among variables make it difficult for traditional analysis methods to obtain more accurate results, while the structural equation model (SEM) can handle the related problems well. Therefore, this study uses Amos software to construct a structural equation model to analyse the relationship between the variables, which is widely used in the research fields of sociology, psychology, economics and behavioural sciences.

SEM is a multivariate statistical analysis method of analysing the relationships between variables based on the covariance matrix between variables, which integrates factor analysis and path analysis. It is mainly divided into a measurement model and a structure model. The measurement model mainly describes the relationships between latent variables and observed variables through factor analysis, while the structural model uses path relationship analysis to measure the relationship between variables. The measurement model is as follows:

X = ∩_x ζ + δ(4)

Y = ∩_y η + ε(5)

Below, ζ is the matrix of the exogenous latent variable, η is the matrix of the endogenous latent variable, Y is the endogenous observation variable, ∩_x represents the load matrix of the exogenous observation variables with respect to the exogenous latent variables. ∩_y represents the load matrix of the endogenous observation variables with respect to the endogenous latent variables. δ represents the measurement error of the exogenous observation variables, and ε is the measurement error of the endogenous observation variables. The structural model is as follows:

η = Bη + Γζ + μ(6)

B represents the coefficient matrix of the relationship between internal latent variables, Γ is the coefficient matrix of the influence of the exogenous latent variables on the endogenous latent variables and μ is the structural vector error.

Firstly, a conceptual model is constructed; then, the parameters of the model are estimated from the survey data to determine the correlation between the variables in the model; finally, the goodness of fit is tested to evaluate whether the model is supported by the actual data.

## 3. Results

### 3.1. The Difference of Happiness among the Urban Young Returnees in China

Table 3 shows that there are significant differences in the happiness of urban young returnees in terms of gender, age, education, time of returning home, current occupation and current monthly income but no significant difference in terms of length of stay in the city and occupation in the city. This suggests that the happiness of urban returnees is influenced more by how they live now in the countryside than by how they used to live in the city.

### 3.2. The Correlations between the Independent Variables and the Happiness of Urban Young Returnees in China

Table 4 indicates that all independent variables are positively associated with urban young returnees’ happiness, and the correlation coefficients are more than 0.6. This shows that the variables we selected are suitable for the next regression.

### 3.3. The Hierarchical Liner Regression Results of Urban Young Returnees’ Happiness

Before applying the structural equation model, hierarchical linear regression is used for the benchmark regression, and the results are shown in Table 5. The variables of social demography, personal factors, institutional factors, social factors are gradually applied to the model. Based on the results of the above multivariate analysis and correlation analysis, we discarded variables such as the duration of residence in the city and occupation in the city because these variables had no significant correlations with the happiness of urban young returnees. The results indicate that the social demography variables, such as gender, monthly income, returning time, current occupation, have no significant effects on the happiness of urban young returnees. Only age and education have positive effects on the happiness of urban young returnees. The results are in contradiction with some previous studies [15,17,18], as urban young returnees are a special group different from common residents. With increasing age and higher education level, more wealth and resources are accumulated, which may improve the sense of happiness.

However, other factors such as career development, policy system, physical environment and urban–rural relationship all have significant positive effects on the happiness of urban young returnees, with the exception of organizational culture, which has a negative impact on urban young returnees’ happiness. Urban young returnees have experienced different cultures in various cities. When they return to rural areas to participate in different organizational cultural activities, there may be short-term collisions and contradictions between the different cultures, which is not conducive to the improvement of their well-being.

### 3.4. The SEM Results of Urban Young Returnees’ Happiness

In order to further analyse the relationships between the independent variables and happiness of urban young returnees, all Q variables are explicit and can be observed directly, and their definitions are shown in Table A1 in the Appendix A. Career development (X1), psychological adaptation (M1), relationship adaptation (M2), collective adaptation (M3) and material adaptation (M4) are used as the exogenous latent variables, and happiness (Y) was taken as the endogenous latent variable to construct a structural equation model. The maximum likelihood method was used to estimate the initial model.

**Figure 2:**
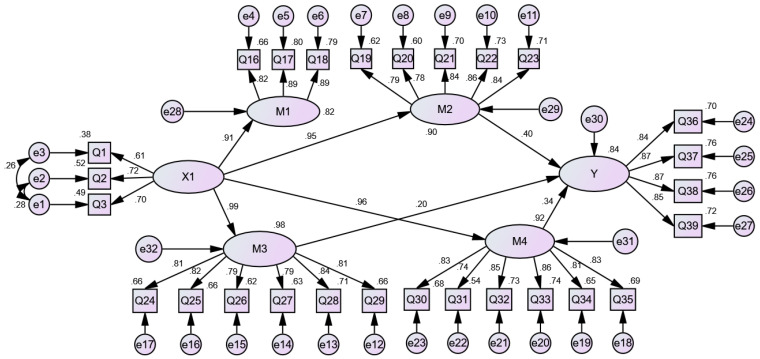
A revised model of the influence mechanism of career development on the happiness of urban young returnees.

**Figure 3:**
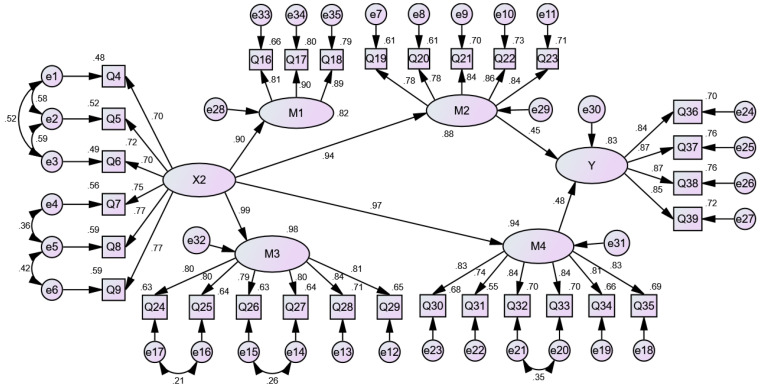
A revised model of the influence mechanism of institutional factors on the happiness of urban returnees.

**Figure 4:**
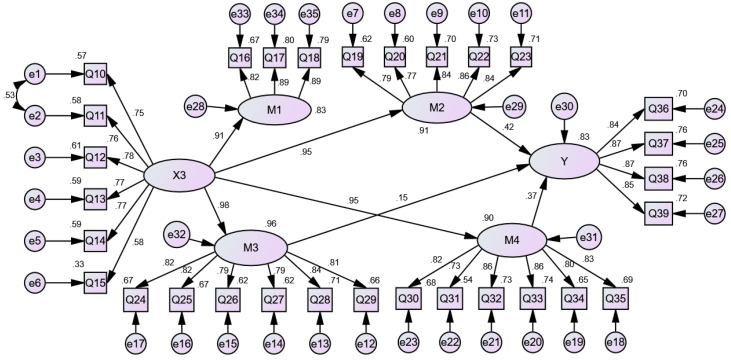
A revised model of the influence mechanism of social factors on the happiness of urban young returnees.

The results of the test of the model fitting parameters show that the *p*-values of two paths, career development → happiness and psychological adaptation (M1) → happiness, are greater than 0.05. Consequently, they are deleted. The *p*-values of the other paths are less than 0.05 and thus statistically significant. The model is modified by the correction index by adding two residual paths [e1–e2] and [e1–e3]. The empirical results show good model fit: relative fit index (CFI) = 0.946, AGFI = 0.867, RMSEA = 0.065. CFI and AGFI exceed the suggested threshold, and RMSEA is lower than the threshold 0.08 (Byrne, 2015). The revised model of the influence mechanism of career development on the happiness of urban returnees is shown in Figure 2. The coefficient connecting the core variables (X1, M1–M4) and the explicit variable Q is the standardized load coefficient, and all coefficients are greater than 0.5, indicating that the model measurement relationship is good. Meanwhile, the coefficient between the latent variables is the standardized path coefficient, which shows the influence relationships between the latent variables. For example, the standardized path coefficient of X1 on M1 is 0.91. The explanation of Figure 3 and Figure 4 is similar to that of Figure 2. Since the significance is not shown in Figure 2, we need to deepen the analysis via Table 6.

To further analyse the influencing mechanism of career development on happiness, the specific path is decomposed as shown in Table 6. From this, we find that career development can significantly affect the material adaptation, collective adaptation, psychological adaptation and relationship adaptation of urban young returnees. Moreover, the standardized indirect effect of Career development → Happiness is 0.901 (*p* < 0.01), and the standardized indirect effect of Career development → Relationship adaptation → Happiness is 0.380 (*p* < 0.01), the standardized indirect effect of Career development → Collective adaptation → Happiness is 0.195 (*p* < 0.01), the standardized indirect effect of Career development → Material adaptation → Happiness is 0.326 (*p* < 0.01). This means that career development does not directly affect the happiness of urban young returnees but indirectly affects their happiness through relationship adaptation, collective adaptation and material adaptation. The indirect effects account for 42.18% (0.380/0.901), 21.64% and 36.18%, respectively. Good career development can expand the network resources and social capital of urban young returnees, leading to the growth of wealth, thus increasing their happiness.

Additionally, the model fitting parameter test results show that the *p*-values of the paths for institutional factors—(X2) → happiness, psychological adaptation (M1) → happiness and collective adaptation (M3) → happiness—are greater than 0.05. Consequently, they are deleted. The *p*-values of the other paths are less than 0.05 and thus statistically significant. The model is modified by the correction index by adding eight residual paths [e1–e2], [e1–e3], [e2–e3], [e4–e5], [e5–e6], [e16–e17], [e14–e15] and [e20–e21]. The empirical results show that the model fit is good: CFI = 0.937, AGFI = 0.859, RMSEA = 0.068. The revised model of the influence mechanism of institutional factors on the happiness of urban returnees is shown in Figure 3.

Table 7 indicates that institutional factors can also affect the material adaptation, collective adaptation, psychological adaptation and relationship adaptation of urban young returnees. In addition, the standardized indirect effect of Institutional factors → Happiness is 0.876 (*p* < 0.01), the standardized indirect effect of Institutional factors → Relationship adaptation → Happiness is 0.410 (*p* < 0.01), and the standardized indirect effect of Institutional factors → Material adaptation → Happiness is 0.466 (*p* < 0.01). This means that institutional factors do not directly affect the happiness of urban young returnees but rather indirectly affect the happiness of urban young returnees through relationship adaptation and material adaptation. The indirect effects accounts for 46.80% (0.410/0.876), 53.20%, respectively. Good systems and policies can create good business environments for urban young returnees; cultivate good business relations; and stimulate entrepreneurship, employment, consumption, etc., thus improving their happiness.

In addition, the model fitting parameter test results show that the *p*-values of social factors (X3) → happiness, psychological adaptation (M1) → happiness are greater than 0.05. Consequently, they are deleted. The *p*-values of the other paths are less than 0.05 and thus statistically significant. The model is modified by the correction index by adding one residual path, [e1–e2]. The empirical results show that the model fit is good: CFI = 0.924, AGFI = 0.824, RMSEA = 0.074. The revised model of the influence mechanism of social factors on the happiness of urban returnees is shown in Figure 4.

Finally, Table 8 indicates that social factors can also affect the material adaptation, collective adaptation, psychological adaptation and relationship adaptation of urban young returnees. The standardized indirect effect of Social factors → Happiness is 0.896 (*p* < 0.01); the standardized indirect effect of Social factors → Relationship adaptation → Happiness is 0.396 (*p* < 0.01); the standardized indirect effect of Social factors → Collective adaptation → Happiness is 0.152 (*p* < 0.01); and the standardized indirect effect of Social factors → Material adaptation → Happiness is 0.348 (*p* < 0.01). This means that social factors do not directly affect the happiness of urban young returnees but indirectly affect the happiness of urban young returnees through relationship adaptation, collective adaptation and material adaptation. The indirect effects account for 44.20% (0.396/0.896), 16.96% and 38.84%, respectively. Social factors are conducive to urban young returnees’ expanding their social resources and social relations and forming collective awareness and social trust, and this cumulative effect will promote their employment, entrepreneurship and consumption, thus increasing their sense of happiness.

## 4. Discussion

The Healthy China 2030 Plan emphasizes that people’s health should be prioritized in development, focusing on rural areas and grass-roots units. The happiness of urban young returnees is also an important form of mental health. The findings in this study differ significantly from those of previous studies. They mainly concentrated on the effects of gender, age, education and marital status on residents’ happiness [16,49]. However, in our study, we focus on urban young returnees, a special group returning to their hometowns or other previous places of residence [6]. Their happiness is affected by career development, institutional factors (especially employment and the entrepreneurship policy system) and social factors (physical environment and the urban–rural relationship). They attach more importance to their career development, and they pay more attention to corporate systems related to their career development than to the household registration system [50]. This implies that today’s urban young returnees have their own unique views and career concepts. Furthermore, urban young returnees focus on the urban–rural relationship. This also contradicts with previous studies [20,25,26] that concentrate on the social relationships. Our study focuses on the urban–rural relationship rather than the social relationships among community residents.

In addition, we introduce the theory of social adaptation to explain the underlying mechanism of urban young returnees’ happiness [44], which includes material adaptation, collective adaptation, relationship adaptation and psychological adaptation [47]. The results of the study do not fully agree with the hypothesis. Another study concludes that career development indirectly affects the happiness of urban young returnees through relationship adaptation, collective adaptation and material adaptation. Institutional factors indirectly affect the happiness of urban young returnees through relationship adaptation and material adaptation. Social factors indirectly affect the happiness of urban young returnees through relationship adaptation, collective adaptation and material adaptation. The mediating effect of psychological adaptation is not significant in the above paths. On the one hand, this shows that psychological adaptation is a long-term process and does not constitute the mechanism of related factors on urban young returnees’ happiness, such as personal factors, institutional factors and social factors. On the other hand, it may be related to the rationality of the preliminary questionnaire design. Despite some limitations, the above mechanisms should generally complement and innovate previous research.

Thus, the findings in this study have important policy implications for improving the well-being of urban young returnees in the context of the Healthy China 2030 Plan. From these data, we can see that the return of urban youth has brought vitality and new changes to their hometowns, which is helpful for rural revitalization and a healthy China. It would be beneficial for the government to promote urban young returnees’ happiness in several aspects. First, a series of measures should be taken such as entrepreneurship and employment policies to encourage urban young returnees to start businesses and gain employment so that they can better adapt to their relationships and material environment and increase their happiness. In addition, the social security system in rural areas should be gradually improved including medical care, pensions, employment and so on. Second, rural infrastructure (roads, facilities, communications, waste disposal) and basic public services (health care and education environment) should be developed. Finally, there should be a breakthrough in the soft environment, for example, promoting integrated and harmonious development between urban and rural areas, strengthening interpersonal relationship and increasing support and encouragement for the urban young returnees from family and society. These can improve their material adaptation, collective adaptation and relational adaptation and thereby enhance their happiness, which in turn will benefit the construction of a healthy China.

This study enriches the literature by highlighting the importance of career development, entrepreneurship and employment policy, and social factors in influencing the happiness of urban young returnees and explaining the underlying mechanisms. However, there are some limitations. First, this paper uses the questionnaire survey method. The main drawback of this method is that we cannot assess the authenticity of the questionnaire completed by the respondents, which may affect the accuracy of the conclusions. In addition, due to the data constraints, only the effects of cross-sectional data are discussed, and the dynamic changes in the happiness of urban young returnees are not considered. Moreover, there may be other paths that affect the happiness of urban young returnees. All of these need to be further explored in the future.

## 5. Conclusions

In the context of the Healthy China 2030 Plan, the happiness of urban young returnees cannot be ignored. Based on a large-scale social practice survey in China, this paper applies a hierarchical linear regression model (HLM) and a structural equation model (SEM) to discuss the influencing factors on the happiness of urban young returnees. The results show that the happiness of urban young returnees in China is less influenced by their socio-demographic characteristics, such as age and education, and more by career development, institutional factors and social factors, which are different from those of ordinary residents. Further study finds that career development indirectly affects the happiness of urban young returnees through relationship adaptation, collective adaptation and material adaptation. The indirect effects account for 42.18%, 21.64% and 36.18% respectively. Institutional factors indirectly affect the happiness of urban young returnees through relationship adaptation (46.80%) and material adaptation (53.20%). Social factors indirectly affect the happiness of urban young returnees through relationship adaptation (44.20%), collective adaptation (16.96%) and material adaptation (38.84%).

## Figures and Tables

**Table 1:** Descriptive information on the respondents.

Characteristics	Classification	Frequency	Rate	Characteristics	Classification	Frequency	Rate
Age	≤20	277	12.6	Living years in the city	3–5	1241	56.4
	21–30	1166	53.0		6–8	290	13.2
	31–40	469	21.3		9–11	132	6.0
	≥41	290	13.2		≥12	539	24.5
Education	Junior high school and below	173	7.9	Returning time	<1	634	28.8
	Senior school or technical secondary school	297	13.5		1–2	463	21.0
	Junior college	398	18.1		2–3	262	11.9
	Undergraduate	1240	56.3		3–4	213	9.7
	Master or above	94	4.3		≥5	630	28.6
Marital status	Unmarried	1263	57.4	Number of Children	0	1348	61.2
	Married	912	41.4		1	514	23.3
	Divorced	25	1.1		2	313	14.2
	Widowed	2	0.1		≥3	27	1.2
Monthly income in the city (yuan)	≤3000	860	39.1	Current monthly income	≤3000	501	22.8
	3001–6000	792	36.0		3001–6000	947	43.0
	6001–9000	358	16.3		6001–9000	495	22.5
	≥9001	192	8.7		≥9001	259	11.8
Occupation in the city	Agriculture, forestry, husbandry and fishery	95	4.3	Current occupation	Agriculture, forestry, husbandry and fishery	189	8.6
	Mining, manufacturing and construction	210	9.5		Mining, manufacturing and construction	192	8.7
	Production and supply of electricity, gas and water	56	2.5		Production and supply of electricity, gas and water	60	2.7
	Transportation, storage and postal services	97	4.4		Transportation, storage and postal services	87	4.0
	Information transmission, computer and software industry	143	6.5		Information transmission, computer and software industry	164	7.4
	Service industry	307	13.9		Service industry	321	14.6
	Financial industry	131	5.9		Financial industry	141	6.4
	Real estate industry	22	1.0		Real estate industry	28	1.3
	Science, education, culture, health and social security	394	17.9		Science, education, culture, health and social security	657	30.7
	Unemployment (including students)	747	33.9		Unemployment (including students)	345	15.7
Gender	Male	987	44.8	Total		2202	100
	Female	1215	55.2				

Source: A large-scale professional social survey conducted by Hangzhou Normal University; the following tables and figures are the same.

**Table 2:** The confirmatory factor analysis results.

Variable	Items ^1^	KMO	Bartlett Spherical Test	Factor Load	α	AVE	CR	Total α
Career development	Q1	0.951	27,742.771	0.828	0.819	0.736	0.893	0.953
	Q2			0.858				
	Q3			0.886				
Organizational culture	Q4			0.917	0.914	0.853	0.946	
	Q5			0.933				
	Q6			0.920				
Policy system	Q7			0.920	0.918	0.859	0.948	
	Q8			0.934				
	Q9			0.927				
Physical environment	Q10			0.924	0.906	0.842	0.941	
	Q11			0.927				
	Q12			0.902				
Urban rural relationship	Q13			0.907	0.801	0.722	0.885	
	Q14			0.918				
	Q15			0.707				
Psychological adaptation	Q16	0.979	40,132.592	0.883	0.898	0.830	0.936	0.972
	Q17			0.930				
	Q18			0.920				
Relationship adaptation	Q19			0.831	0.909	0.736	0.933	
	Q20			0.820				
	Q21			0.868				
	Q22			0.893				
	Q23			0.876				
Collective adaptation	Q24			0.839	0.920	0.716	0.938	
	Q25			0.854				
	Q26			0.843				
	Q27			0.837				
	Q28			0.871				
	Q29			0.833				
Material adaptation	Q30			0.841	0.923	0.725	0.940	
	Q31			0.788				
	Q32			0.879				
	Q33			0.889				
	Q34			0.842				
	Q35			0.865				
Happiness	Q36			0.867	0.915	0.799	0.941	
	Q37			0.904				
	Q38			0.911				
	Q39			0.892				

Note: all load values are significant at 0.001. α is the internal consistency reliability coefficient. CR is the combination reliability coefficient. AVE is the average amount of variance extracted. ^1^ Detailed information are presented in Table A1 in the Appendix A.

**Table 3:** The differences in happiness among urban young returnees in China.

Variable	Classification	Happiness	T(P)/F(P)
Gender	Male	15.24 ± 3.73	2.223 (0.026)
	Female	14.90 ± 3.34	
Age	≤20	14.40 ± 3.97	7.050 (<0.001)
	21–30	14.93 ± 3.54	
	31–40	15.45 ± 3.27	
	≥41	15.49 ± 3.27	
Education	Junior high school and below	14.23 ± 3.46	3.384 (0.009)
	Senior school or technical secondary school	14.95 ± 3.89	
	Junior college	14.97 ± 3.47	
	Undergraduate	15.18 ± 3.41	
	Master or above	15.55 ± 3.52	
Marital status	Unmarried	14.93 ± 3.64	1.835 (0.139)
	Married	15.21 ± 3.36	
	Divorced	15.36 ± 3.09	
	Widowed	11.50 ± 2.12	
Number of Children	0	14.94 ± 3.61	1.422 (0.234)
	1	15.27 ± 3.34	
	2	15.20 ± 3.36	
	≥3	14.70 ± 4.26	
Length of stay in the city	3–5	15.11 ± 3.49	1.034 (0.376)
	6–8	15.00 ± 3.34	
	9–11	15.37 ± 3.49	
	≥12	14.86 ± 3.71	
Returning time	<1	14.63 ± 3.91	4.393 (0.002)
	1–2	15.21 ± 3.17	
	2–3	14.89 ± 3.60	
	3–4	15.62 ± 3.00	
	≥5	15.23 ± 3.45	
Occupation in the city	Agriculture, forestry, husbandry and fishery	14.83 ± 4.17	0.980 (0.455)
	Mining, manufacturing and construction	15.30 ± 3.75	
	Production and supply of electricity, gas and water	14.86 ± 3.78	
	Transportation, storage and postal services	14.92 ± 3.69	
	Information transmission, computer and software industry	15.48 ± 3.03	
	Service industry	14.96 ± 3.42	
	Financial industry	15.27 ± 3.63	
	Real estate industry	16,45 ± 2.46	
	Science, education, culture, health and social security	14.97 ± 3.40	
	Unemployment (including students)	14.95 ± 3.53	
Current occupation	Agriculture, forestry, husbandry and fishery	14.77 ± 4.28	2.934 (0.002)
	Mining, manufacturing and construction	15.64 ± 3.32	
	Production and supply of electricity, gas and water	14.58 ± 3.87	
	Transportation, storage and postal services	14.86 ± 3.64	
	Information transmission, computer and software industry	15.47 ± 3.14	
	Service industry	14.96 ± 3.41	
	Financial industry	14.66 ± 3.67	
	Real estate industry	15.79 ± 2.97	
	Science, education, culture, health and social security	15.31 ± 3.13	
	Unemployment (including students)	14.48 ± 3.94	
Monthly income in the city (yuan)	≤3000	14.84 ± 3.62	1.733 (0.158)
	3001–6000	15.17 ± 3.21	
	6001–9000	15.27 ± 3.42	
	≥9001	15.08 ± 4.39	
Current monthly income	≤3000	14.37 ± 3.86	9.80 (<0.001)
	3001–6000	15.10 ± 3.15	
	6001–9000	15.53 ± 3.36	
	≥9001	15.27 ± 4.18	

**Table 4:** The correlations between the core variables and happiness.

Variables	Dimensions	SWB ^1^
Personal factors	Career development	0.724 ***
Institutional factors	Organizational culture	0.601 ***
	Policy system	0.661 ***
Social factors	Physical environment	0.707 ***
	Urban rural relationship	0.716 ***
Psychological adaptation	Self-identity	0.668 ***
	Self-efficacy	0.689 ***
	Self-expectation	0.709 ***
Relationship adaptation	Genetic relationship	0.746 ***
	Geographical relation	0.786 ***
	Business relationship	0.707 ***
Collective adaptation	Living environment	0.765 ***
	Social trust	0.708 ***
	Social networks	0.776 ***
Material adaptation	Consumption pattern	0.754 ***
	Employment structure	0.760 ***
	Social security	0.752 ***

Note: *** represent significance at the 1% level. ^1^ SWB: Subjective well-being.

**Table 5:** Hierarchical linear regression results for urban young returnees’ happiness.

Variable	Tier 1 ^1^	Tier 2 ^2^	Tier 3 ^3^
	Beta	Beta	Beta
Gender (Female)			
Male	−0.050 *	−0.013	−0.017
Age (Aged below 20)			
Aged between 21 and 30	0.018	0.019	0.006
Aged between 31 and 40	0.093 *	0.071 **	0.047 *
Aged over 41	0.122 **	0.072 **	0.042 *
Education (Junior high school and below)			
Senior school or technical secondary school	0.112 **	0.039	0.023
Junior college	0.152 **	0.030	0.022
Undergraduate	0.250 **	0.064 *	0.046 *
Master or above	0.112 **	0.037 *	0.034 *
Returning time (Less than 1 year)			
Between 1 and 2 years	0.046	0.009	0.011
Between 2 and 3years	0.004	−0.012	−0.010
Between 3 and 4 years	0.062 **	0.011	−0.010
More than 5 years	0.014	−0.032	−0.026
Current occupation (Agriculture, forestry, husbandry and fishery)			
Mining, manufacturing and construction	0.059 *	0.015	−0.001
Production and supply of electricity, gas and water	−0.020	0.002	−0.010
Transportation, storage and postal services	0.005	0.005	−0.016
Information transmission, computer and software industry	0.029	0.018	−0.008
Service industry	0.010	−0.010	−0.017
Financial industry	−0.022	−0.002	−0.019
Real estate industry	0.023	−0.004	−0.003
Science, education, culture, health and social security	0.031	0.024	−0.003
Unemployment	0.032	0.011	0.013
Current Monthly income (≤3000)			
3001–6000	0.083 *	0.001	0.007
6001–9000	0.096 *	0.004	0.007
≥9001	0.032	−0.008	−0.001
Personal factors			
Career development		0.306 **	0.161 **
Institutional factors			
Organizational culture		−0.035	−0.081 **
Policy system		0.121 **	0.017
Social factors			
Physical environment		0.210 **	0.020
Urban rural relationship		0.304 **	0.061 **
Psychological adaptation			
Self-identity			0.006
Self-efficacy			−0.025
Self-expectation			0.080 **
Relationship adaptation			
Genetic relationship			0.031
Geographical relation			0.186 **
Business relationship			0.034
Collective adaptation			
Living environment			0.089 **
Social trust			0.008
Social networks			0.098 **
Material adaptation			
Consumption pattern			0.092 **
Employment structure			0.116 **
Social security			0.107 **
R2	0.205	0.812	0.877
F	3.996	144.653	175.590
ΔR2	0.042	0.617	0.110
ΔF	3.996	785.258	86.064
VIFmax	4.903	5.022	5.093

Note: ** and * represent significance at the 5% and 10% levels, respectively. ^1^ Tier 1 refers to the social demography variables input into the model. ^2^ Tier 2 refers to the social demography, personal factor, institutional factor and social factor variables input into the model. ^3^ Tier 3 refers to all the control variables input into the model.

**Table 6:** The effects of career development on the happiness of urban young returnees.

Paths	Standardized Direct Effect	Standardized Indirect Effect	Standardized Total Effect	*p*
Career development → Psychological adaptation	0.907		0.907	***
Career development → Relationship adaptation	0.949		0.949	***
Career development → Collective adaptation	0.989		0.989	***
Career development → Material adaptation	0.960		0.960	***
Psychological adaptation → Happiness	/		/	/
Relationship adaptation → Happiness	0.400		0.400	***
Collective adaptation → Happiness	0.197		0.197	***
Material adaptation → Happiness	0.340		0.340	***
Career development → Happiness	/	0.901	0.901	***
Career development → Psychological adaptation → Happiness	/	/	/	/
Career development → Relationship adaptation → Happiness		0.380	0.380	***
Career development → Collective adaptation → Happiness		0.195	0.195	***
Career development → Material adaptation → Happiness		0.326	0.326	***

Note: *** represent significance at the 1% level.

**Table 7:** The effects of institutional factors on the happiness of urban young returnees.

Paths	Standardized Direct Effect	Standardized Indirect Effect	Standardized Total Effect	*p*
Institutional factors → Psychological adaptation	0.904	/	0.904	***
Institutional factors → Relationship adaptation	0.938	/	0.938	***
Institutional factors → Collective adaptation	0.989	/	0.989	***
Institutional factors → Material adaptation	0.967	/	0.967	***
Psychological adaptation → Happiness	/	/	/	/
Relationship adaptation → Happiness	0.453		0.453	***
Collective adaptation → Happiness	/	/	/	***
Material adaptation → Happiness	0.482	/	0.482	***
Institutional factors → Happiness	/	0.876	0.876	***
Institutional factors → Psychological adaptation → Happiness	/	/	/	/
Institutional factors → Relationship adaptation → Happiness	/	0.410	0.410	***
Institutional factors → Collective adaptation → Happiness	/	/	/	***
Institutional factors → Material adaptation → Happiness	/	0.466	0.466	***

Note: *** represent significance at the 1% level.

**Table 8:** The effects of social factors on the happiness of urban young returnees.

Paths	Standardized Direct Effect	Standardized Indirect Effect	Standardized Total Effect	*p*
Social factors → Psychological adaptation	0.909		0.909	***
Social factors → Relationship adaptation	0.954		0.954	***
Social factors → Collective adaptation	0.978		0.978	***
Social factors → Material adaptation	0.949		0.949	***
Psychological adaptation → Happiness	/	/	/	/
Relationship adaptation → Happiness	0.415		0.415	***
Collective adaptation → Happiness	0.155		0.155	***
Material adaptation → Happiness	0.367		0.367	***
Social factors → Happiness	/	0.896	0.896	***
Social factors → Psychological adaptation → Happiness	/	/	/	/
Social factors → Relationship adaptation → Happiness	/	0.396	0.396	***
Social factors → Collective adaptation → Happiness	/	0.152	0.152	***
Social factors → Material adaptation → Happiness	/	0.348	0.348	***

Note: *** represent significance at the 1% level.

## Data Availability

Restrictions apply to the availability of these data because the data were obtained from a third party. They can be made available by the first authors (F.-W.S. and J.Z.) with the permission of the third party.

## Appendix A

**Table A1:** The detailed items.

Variable	Items	
Career development	Q1 Always pay attention to and search for the information about the occupation I am interested in.	
	Q2 The countryside provides me with a stage to give full play to my professional expertise.	
	Q3 I am optimistic about the future of my current job.	
Organizational culture	Q4 My village often holds cultural activities (village evening party, writing Spring Festival couplets, comparison of filial piety and morality, etc.).	
	Q5 My village often carries out cultural publicity activities (lectures, broadcasting, door-to-door publicity, etc.).	
	Q6 My village has various cultural organizations (Yangko team, square dance team, band, etc.).	
Policy system	Q7 The village where I live lets young urban returnees know about relevant employment and entrepreneurship policies through various channels.	
	Q8 The village where I live has supportive policies for entrepreneurship and employment.	
	Q9 The village where I live has policies to attract young people from the cities to return to their hometowns to find jobs and start businesses.	
Physical environment	Q10 My village has complete infrastructure (roads, facilities, communications, waste disposal).	
	Q11 The basic public service system (medical and health, education environment) in my village is sound.	
	Q12 The economic development of my village is better.	
Urban rural relationship	Q13 I prefer the way of life in the countryside.	
	Q14 I prefer the pace of life in the countryside.	
	Q15 I think there are development differences between urban and rural areas.	
Psychological adaptation	Self-identity	Q16 I’m proud of being a rural person.
	Self-efficacy	Q17 In the countryside, I can give full play to my ability.
	Self-expectation	Q18 In the countryside, I can achieve my goals and ideals.
Relationship adaptation	Genetic relationship	Q19 My family and friends agreed with my choice of returning home.
		Q20 My family and friends have given me funds and contacts to start my business.
	Geographical relationship	Q21 I am willing to take part in the cultural construction or cultural activities of the village.
		Q22 I live in a harmonious village.
	Business relationship	Q23 I prefer the way I get along with people when I work in the countryside.
Collective adaptation	Living environment	Q24 I think the living environment is good now.
		Q25 I think the living standard is high now.
	Social trust	Q26 The public service expenditure in my village is high.
		Q27 My village has high financial transparency.
	Social networks	Q28 It’s easy for me to get help from others or organizations.
		Q29 I have established close relationships with potential or existing friends.
Material adaptation	Consumption pattern	Q30 I think the proportion of basic living expenses in the total income is reasonable.
		Q31 I agree with the consumption mode that attaches importance to material life.
	Employment structure	Q32 I understand the social security system (medical, pension, employment, etc.) in my village.
		Q33 I am satisfied with the social security system (medical, pension, employment, etc.) in my village.
	Social security	Q34 I think the quality of the workers in the village is high.
		Q35 I think the non-agricultural industry in my village has absorbed more workers.
Happiness	Q36 My life is close to my ideal in many ways.	
	Q37 My life goal is enough to give me the motivation to struggle.	
	Q38 I feel happy when I devote myself to what I do.	
	Q39 I think my work can bring positive influence to the development of my hometown.	
